# A case report of acute promyelocytic leukemia with myeloid sarcoma of the lumbar spine and literature review

**DOI:** 10.3389/fmed.2024.1507716

**Published:** 2025-01-22

**Authors:** Yiwen Du, Kun Yang, Yantao Ling, Ying Zhang, Yuping Gong

**Affiliations:** West China Hospital, Sichuan University, Chengdu, China

**Keywords:** acute promyelocytic leukemia, myeloid sarcoma, extramedullary infiltration, literature review, treatment

## Abstract

Acute promyelocytic leukemia (APL) presenting solely as myeloid sarcoma (MS) is extremely rare. This report describes a 53-year-old male who presented with low back pain and a movement disorder in his lower limbs. MRI and PET/CT scans of the lumbar spine revealed an intraspinal mass. Pathological analysis of the surgically resected mass identified it as myeloid in origin. Routine blood tests were unremarkable, and bone marrow smears and immunophenotyping showed no evidence of abnormal myeloblasts or promyelocytes. However, bone marrow aspirates testing for acute leukemia fusion genes by qPCR revealed the presence of the *PML::RARA* fusion. Further investigation via FISH confirmed the fusion in both the bone marrow and the extramedullary mass. The patient was ultimately diagnosed with isolated promyelocytic extramedullary sarcoma (MS/APL). Treatment with all-trans retinoic acid and arsenic trioxide alleviated the back pain and restored the patient’s mobility. After 1 year of consolidation therapy, bone marrow smears confirmed sustained remission, and the *PML::RARA* fusion gene was undetectable. In addition to this case, we review 41 other APL patients with extramedullary sarcoma as their first symptom (MS/APL) at the time of diagnosis and provide an analysis of these cases.

## Introduction

Acute promyelocytic leukemia (APL) is a subtype of acute myeloid leukemia (AML) defined by the genetic translocation that forms the *PML::RARA* fusion gene between chromosomes 15 and 17 (1). This fusion disrupts gene transcription, halting myeloid differentiation at the promyelocytic stage (1, 2). APL accounts for approximately 10%–15% of all AML cases and is typically diagnosed through abnormal blood tests, along with coagulation and fibrinolytic dysfunction (3, 4). The incorporation of all-trans retinoic acid (ATRA) and arsenic trioxide (ATO) into treatment protocols has dramatically improved outcomes for APL patients, achieving a complete remission (CR) rate exceeding 90% (5, 6).

Myeloid sarcoma (MS), also known as granulocytic sarcoma or chloroma, is characterized by the extramedullary accumulation of myeloid blasts (7). It can occur as an isolated condition or in association with myeloid malignancies, particularly AML, and often signifies relapse following AML remission (7, 8). Although MS can affect individuals of all ages, it is more common in children than adults, with a male-to-female ratio of approximately 1.2:1 (7, 9, 10). MS/extramedullary infiltration is a rare complication of APL, affecting only 3%–5% of patients, typically during disease relapse post-remission (11, 12). The central nervous system and skin are the most common sites of extramedullary involvement, while other areas such as lymph nodes, the gastrointestinal tract, bones, soft tissues, and testes are less frequently affected (12, 13). Rare cases of APL-related EM infiltration at unusual sites have also been reported. Key factors associated with extramedullary involvement in APL include being under 45 years of age, elevated white blood cell count, and the presence of the bcr3 subtype of the *PML::RARA* fusion gene (14). The occurrence of APL with MS or EM infiltration as the sole initial presentation is extremely rare. Here, we present a case of APL-related MS manifesting as a lumbar epidural mass.

## Case presentation

**FIGURE 1:**
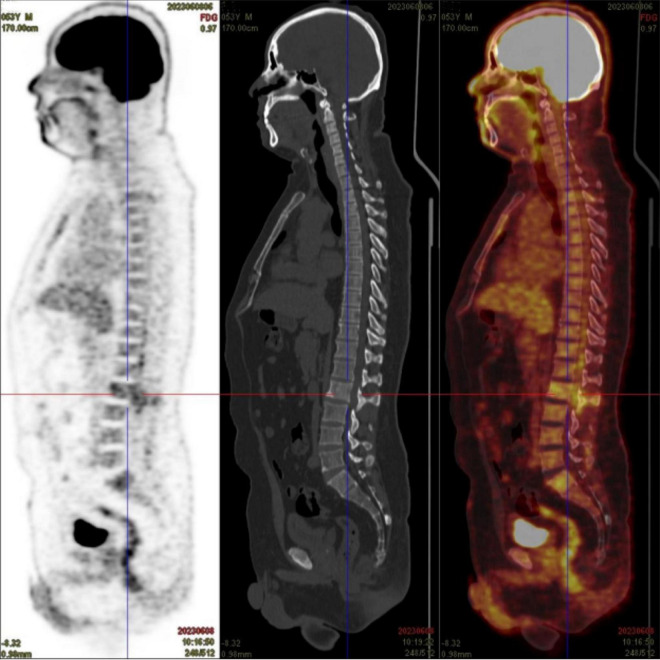
The PET/CT scan of the patient showed areas of uneven density in several vertebrae. Slightly increased FDG uptake was observed in the vertebral bodies and appendages, with active FDG metabolism also detected in the spinal cord cavity between the T12 and L2 segments.

A 53-year-old male presented with 8 months of low back pain and weakness in both lower limbs. A CT scan at a local hospital revealed soft tissue shadows at the right posterior margin of the L2/3 intervertebral disc and in the spinal canal at the same level. MRI showed abnormal signals in the T12, L2, and S1 vertebral bodies, along with intraspinal soft tissue masses at the L2 pyramidal plane. Neoplastic lesions were suspected, and the patient received treatment with traditional Chinese medicine. Although there was initial improvement, his condition progressively worsened, leading to an inability to walk. PET/CT scans revealed uneven density in several vertebrae, with soft tissue shadows in the right portion of the L2 vertebra, extending into the right intervertebral foramen and inward into the spinal canal. There was slightly increased FDG uptake in the vertebral bodies and appendages, and active FDG metabolism was also noted in the spinal cord cavity from the T12-L2 segment (Figure 1). A follow-up MRI 1 month later showed multiple areas of bone destruction in the T2, L1, L2, and S1-3 vertebrae, suggesting metastatic involvement. Additionally, heterogeneous signal intensity in the spinal canal at the L1-3 level indicated possible involvement. The patient underwent surgical resection of the L2 vertebral body and the epidural mass, along with spinal fixation. Preoperative blood tests, including routine examinations and coagulation studies, were normal. Histopathological analysis of the resected tissue suggested a neoplastic tumor.

**FIGURE 2:**
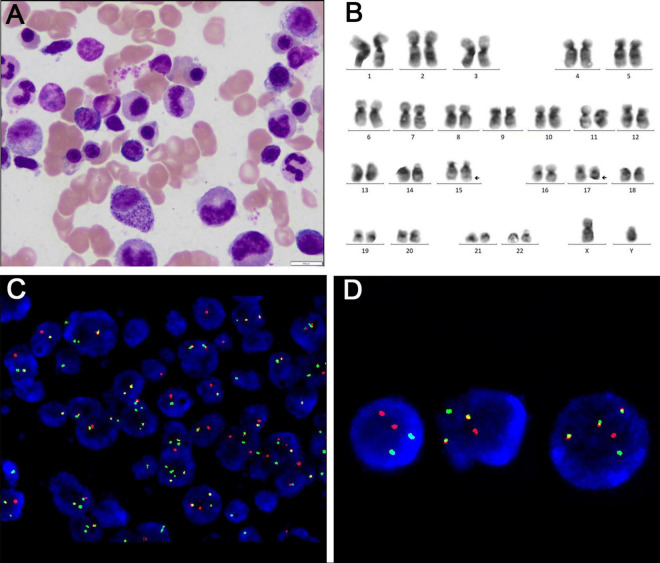
**(A)** Bone marrow cytomorphology by bone marrow aspirate smear. **(B)** Karyotype of bone marrow. **(C)** FISH probe detection of myeloid sarcoma biopsies. **(D)** FISH probe detection of bone marrow aspirate.

The case was referred to our pathology department for further consultation. Immunophenotyping results were as follows: CD34 (−), CD117 (+), MPO (+), CD20 (−), CD79a (−), CD3 (−), CD138 (−), CD38 (−), Mum-1 (−), CD56 (−), IgK (−), Igλ (−), and Ki-67 (+, approximately 60%). *In situ* hybridization for EBV showed no EBER1/2 expression. Gene rearrangement analysis by PCR and GENESCAN revealed no clonal amplification peaks for IgH or IgK. Based on these results, along with the morphological and immunophenotypic findings, MS was strongly considered. Postoperatively, the patient showed some improvement in low back pain and lower limb weakness, but remained unable to stand or walk. One month after surgery, the patient sought treatment at our hematology clinic. A bone marrow smear revealed significantly active marrow hyperplasia, but no blasts or abnormal promyelocytes were detected (Figure 2A). Flow cytometry showed abnormal promyelocytes with approximately 0.5% of nucleated cells, positivity for CD123, CD9, CD117, CD64, and CD33, but negativity for HLA-DR, CD11b, CD15, and CD56. These findings raised strong suspicion for APL-associated MS. Further tests confirmed our suspicion: PCR of peripheral blood was positive for *PML::RARA*, with a *PML::RARA*/*ABL* ratio of 0.9305%. Chromosomal analysis revealed 46,XY,t(15;17)(q24;q21)[2]/46,XY[18] (Figure 2B). Multiplex real-time PCR of bone marrow also showed positivity for *PML::RARA* (bcr-1). FISH analysis of the MS tissues revealed a 94% positivity rate for *PML::RARA* fusion signals (Figure 2C), while bone marrow FISH showed a 8% positivity rate for *PML::RARA* fusion at the 17q21/15q22-24 site, including 4% atypical signals (Figure 2D). Genetic testing revealed no mutations typically associated with AML prognosis at diagnosis. Routine blood tests, coagulation, and fibrinolysis remained normal, with no hepatosplenomegaly or systemic lymphadenopathy observed. Given these findings, the patient was diagnosed with acute promyelocytic extramedullary sarcoma (MS/APL).

**FIGURE 3:**
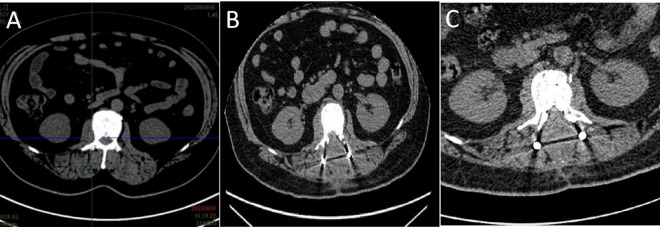
**(A)** PET/CT scan at the L2 level before surgery. **(B)** CT scan at the L2 level after surgery. **(C)** CT scan at the L2 level after one cycle of retinoic acid combined with arsenic trioxide induction therapy.

**FIGURE 4:**
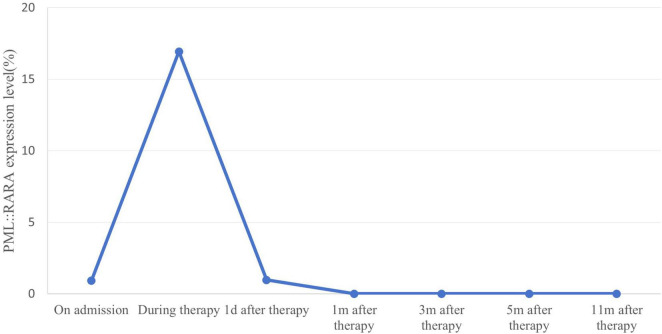
Quantitative changes in expression of *PML::RARA* fusion gene monitored by real-time PCR. *PML::RARA* fusion gene levels in peripheral blood of patients were dynamically monitored by real-time PCR. The detected value of *PML::RARA* level (*PML::RARA/ABL*) = *PML::RARA* copy number/*ABL* copy number × 100%. When the detected value showed negative, it indicated that there was no fusion gene expression or the fusion gene expression level in the submitted samples was lower than the lower limit of detection (100 copies/ml) of this method. The *PML::RARA* fusion gene decreased after chemotherapy and remained negative during maintenance therapy.

The patient began treatment on the 48th day post-surgery, consisting of ATRA 10 mg three times daily and ATO 10 mg intravenously once a day for 30 days. During treatment, the patient developed mild ATRA syndrome, including fever, facial edema, and weight gain, which were managed with dexamethasone and furosemide. In addition, the expression level of the *PML::RARA* fusion gene reached the highest value on day 24 of induction treatment with a *PML::RARA/ABL* ratio of 16.3309%. After the first cycle of chemotherapy, the patient’s condition improved significantly. He was able to stand and walk independently with a brace, and his lumbar pain was greatly reduced. Bone marrow smears revealed no blasts, with promyelocytes comprising 1% of nuclear cells. Both peripheral white blood cell and blood cell counts normalized, indicating CR. However, the *PML::RARA* fusion gene remained detectable, with a *PML::RARA/ABL* ratio of 0.9669%. Following discharge, the patient continued ATRA at 10 mg three times daily, alternating with 2 weeks of rest, followed by 2 weeks of Realgar-Indigo naturalis formula (RIF, 5 tablets three times daily). This consolidation therapy was planned for two cycles. Two months later, a bone marrow smear confirmed sustained CR, and the *PML::RARA* fusion gene was no longer detectable. CT scans of the lumbar spine showed no mass (Figure 3). The patient’s back pain had significantly improved, and he was able to walk freely. The consolidation regimen of ATRA and RIF was maintained for 6 months, with regular monitoring of bone marrow cytology, genetic tests, and spinal imaging every 2 months, all of which showed normal results. Eight months after the first induction, the patient received radiation therapy. He tolerated the treatment well with no major complications. One year after treatment, the patient remained in remission, with molecular analyses of bone marrow and peripheral blood showing no evidence of *PML::RARA* fusion transcripts (Figure 4).

## Literature review

Our review identified 41 cases of APL where MS was the initial presenting symptom. Key details of these cases, including onset locations, clinical features, and treatment responses, are summarized in Table 1 (15–54). The patients were predominantly young, with a median age of 39.5 years (range: 1–77 years), and only 15% were aged ≥60 years. The male-to-female ratio was 24:17, showing no significant sex differences. The spine was the most common site of extramedullary infiltration (12/41 cases) (15, 18, 27, 28, 33, 35, 37, 43, 49–51, 54), followed by the skin (4/41) (19, 29, 42, 52), pleura (3/41) (19, 40, 53), and ovary (2/41) (25, 49). Other less common sites included the intracranial region (2/41) (22, 48), tongue (2/41) (24, 44), humerus (2/41) (23, 39), and colon (2/41) (31, 46), among others. Notably, one patient developed sarcoma in a donor kidney after renal transplantation, not in their own kidney (45). Most MS cases were confined to a single site (78%, 32/41), with multiple-site (9 cases) and multi-organ (7 cases) involvement occurring less frequently. Immunophenotyping of extramedullary masses typically showed MPO positivity. Other markers included CD68 (20%, 8/41), CD43, CD33, and CD117 (15%, 6/41), and CD13 (10%, 4/41). Bone marrow infiltration was observed in 59% (24/41) of patients, while 42% (17 patients) (17, 19, 21, 23, 25, 26, 32, 33, 37–39, 41, 43, 45, 49, 53, 54) had no blasts or promyelocytes in the bone marrow and circulating blood, or did not meet the diagnostic criteria for APL. Six patients had elevated white blood cell counts (16, 22, 24, 41, 42, 44), and three presented with disseminated intravascular coagulation (DIC) (15, 22, 53). Based on white blood cell counts, patients were classified into high-risk (6 patients) (16, 22, 24, 41, 42, 44) and low-risk (27 patients) (15, 17–20, 25–31, 34–40, 46–53) groups, while the remaining cases (21, 23, 32, 33, 43, 54) could not be classified. Chromosome 15 and 17 translocations (t(15;17)) were detected in 54% (22/41) of cases. Seven cases (17%) (22, 25, 33, 41, 47, 49, 52) had a normal karyotype, and 5% (2/41) (48, 54) had complex karyotypes. The common *PML::RARA* fusion was present in 59% (24/41) of patients, while rare fusion signals involving *RARA* [fused with *NPM1* (35), *FIP1L1* (48), *ZBTB16* (51), and *TTMV* (54)] were detected in four cases. One case lacked *RARA* rearrangement, but RT-PCR testing revealed an in-frame fusion between *CPSF6* exon 4 and *RARG* exon 4 (*CPSF6:: RARG*) (47). Six patients (15%) had concurrent gene mutations, with *FLT3* mutations being the most common (7%, 3/41) (37, 41, 50). Other mutations included *KARS* (47, 48) and *WT1* (47, 54) (each in two patients), as well as *EZH2* (47), *KMT2C* (50), and *SMAD9* (54) mutations.

**TABLE 1:** Cases of acute promyelocytic leukemia with MS as the first symptom.

Reference	Age/ sex	MS site	Type	Immuno-phenotype	High WBC count	DIC	Blasts/ promy-elocyte in BM (%)	Auer rods	Karyo type	Fusion gene	Gene mutation	Risk level	Treat-ment	Response	PFS
Blesco et al. (16)	4/M	Pelvis	Single	NA	+	−	−/59	NA	NA	NA	NA	Adverse	Vincristine + prednisone + adriamycin, radiotherapy	NR	-(CR after 14 m)
Kubonishi et al. (17)	23/M	Mediastinum	Single	MPO	−	−	NA/NA (2 m later: 3/63)	−	NA	NA	NA	Favorable	Radiotherapy, mediastinal tumor resection,	NR	-(14 m later died of heart failure)
Zuiable et al. (18)	31/M	(T12∼L3/4) extradural space	Multiple	NA	−	−	2/NA NA/90	NA	NA	NA	NA	Favorable	Laminectomy, radiotherapy, DA, atuo-HSCT	CR	>18 m
Tosi et al. (15)	27/M	(L3∼4) extradural space	Multiple	MPO, CD43, Lys	−	+	−/NA (many of promyelocyte cells)	+	t(15;17) (q22;q11)	NA	NA	Favorable	Laminectomy, ATRA + DA	NR	–
Bobbio-Pallavicini et al. (19)	–/M	Pleura, fronto-parietal scalp and lumbar region	Multiple	MPO, CD43	−	−	−/−	−	NA	*PML::RARA* (bcr1)	NA	Favorable	Chemotherapy (involve ATRA)	CR	13 m
Takeh et al. (20)	66/M	Ilem	Single	NA (infiltration by giant promyelocytes)	−	−	NA/infiltration by giant promyelocytes	NA	NA	NA	NA	Favorable	Limited intestinal resection and anastomosis	NA	Died 14 h after surgery
Gopal et al. (21)	27/M	Left testicle	Single	MPO, CD43, CD117, CD33, CD34	NA	NA	−/−	NA	46,XY,t(15;17) (q22.3;q21.1) [ 15]	NA	NA	NA	Radical orchiectomy	NA	12 m (and relapsed in the contrala-teral testicle)
Fukushima et al. (22)	39/F	Left cerebellar hemisphere	Single	LCA, CD13, CD33	+	+	NA/NA (with proliferation of abnormal promyelocytes)	+	Normal	*PML::RARA*	NA	Adverse	IA, Posterior fossa decompression	NA	1 m (died 4 days after surgery)
Worch et al. (23)	16/F	Right humerus, right proximal femur, and distal tibia	Multiple	MPO, CD13, CD15, CD33, CD117	NA	NA	−/−	−	t(15;17)	*PML::RARA*	NA	NA	ATRA	CR	NA
Mohamedbhai et al. (24)	45/M	Tongue	Single	MPO, CD45, CD68	+	−	NA/NA (diffuse infiltration)	NA	t(15;17) (q22;q12)	NA	NA	Adverse	ATRA + DA	CR	>1 m
Wang et al. (25)	26/F	Right ovary	Single	MPO, TdT, CD13, CD33, CD99, CD45 (LCA), CD20, CD3, CK, Vim, INH, PLAP	−	−	−/−	−	Normal	*PML::RARA*	NA	Favorable	IA, MA, 6-MP + MTX + ATRA	CR	27 m and then progressed to AML with t(8;21) (q22;q22)/ RUNX1:: RUNX1T1 (FAB type:M2)
Thomas and Chelghoum (26)	19/M	Sternum	Single	NA	−	−	−/−	−	t(15;17) (q22;q21-22)	*PML::RARA*	NA	Favorable	Tumor resection, ATRA + IA, radiotherapy	CR	>24 m
Kyaw et al. (27)	26/M	(T2∼4, T12∼L2) extradural space	Multiple	NA	−	−	NA/NA (diffuse infiltration)	NA	NA	*PML::RARA* (bcr1)	NA	Favorable	Radiotherapy, ATRA + DNR	CR	>5 m
Bittencourt et al. (28)	53/M	(T6∼T8) extradural space	Multiple	NA	−	−	NA/NA (diffuse infiltration)	+	46,XY,t(15;17) (q22;q12)	*PML::RARA*	NA	Favorable	ATRA + DNR, radiotherapy	CR	NA (soon after hematological remission) and died of sepsis
Shvartsbeyn et al. (29)	46/M	Abdominal skin	Multiple	NA (myeloid nature)	−	−	∼95/NA	+	t(15;17)	*PML::RARA*	NA	Favorable	ATRA + IDA + dexamethasone	Dead	Died of multi-organ failure
Benjazia et al.(30)	17/F	Rectum	Single	MPO, Lys, CD43	−	−	80/NA	+	46,XX,t(15;17) (q22;q21)	*PML::RARA*	NA	Favorable	ATRA + IDA	CR	>48 m
Damodar et al. (31)	29/M	Colon	Single	MPO, CD43, CD3, Ki67 (70%)	−	−	NA/NA (reported as AML)	NA	t(15;17) (q24;q21)	*PML::RARA*		Favorable	ATRA + DNR	CR	NA
Yamashita et al. (32)	1/M	Mandible	Single	CD45	NA	−	NA/NA	NA	NA	*PML::RARA*	NA		ATRA + anthracycline antitumor agent	CR	>12 m
Piñán et al. (33)	61/F	(T12∼L1) extradural space	Multiple	MPO, CD43, CD68, Ki67	NA	NA	−/−	−	Normal	–	NA	NA	Laminectomy, radiotherapy	NA	Progre-ssion to APL after 9 months
Li et al. (34)	44/M 31/F	(Left 3rd, right 4th) costal cartilage; Perianal	Multiple	MPO, MPO, Vim, LCA, CD3, CD5, CD20, Actin, CD2, Kappa, Lambda, S-100, Ki67 (∼50%)	−*CPSTABLEENTER*−	−*CPSTABLEENTER*−	23/55; 3/92	NA	NA NA	*PML::RARA* (bcr3) *PML::RARA* (bcr1)	NA NA	Favorable Favorable	ATRA + ATO + THP; ATRA + ATO + THP + AraC	CR CR	>24 m >24 m
Kikuma et al. (35)	52/M	(The 7th thoracic) vertebra	Single	MPO, CD68, Lys	−	−	89.2/NA	−	46,XY,t(5;17) (q35;q12)	*NPM1::RARA*	NA	Favorable	Steroid, radiotherapy, IA + ATRA	CR	NA
Rodriguez et al. (36)	43/F	Appendix	Single	MPO, CD68	−	−	90/NA	NA	t(15;17) (q22;q12)	*PML::RARA*	–	Favorable	Laparoscopic appendectomy, ATRA + DA	CR	NA
Shah et al. (37)	56/M	Extradural space	Multiple	MPO, CD43, CD45, CD68, CD117	−	−	NA/NA	−	46,XY,t(15;17) (q24;q21) [ 16]/46,XY[ 5]	*PML::RARA*	FLT3-ITD	Favorable	T5-T9 decompressive laminectomy with fusion and resection of the epidural mass, ATRA + IDA	CR	12 m (and developed relapse periphe-rally)
de Andrade et al. (38)	24/F	Oral cavity	Single	MPO, CD99, Ki67 (60%)	−	−	NA/NA	NA	t(15;17)	NA	NA	Favorable	ATRA + IA	NA	1 m (and died of hemorrh-age)
Sawhney et al. (39)	52/F	Right humerus	Single	MPO, CD33, CD117, CD71, CD34	−	−	−/−	NA	t(15;17)	*PML::RARA*	NA	Favorable	ATRA + ATO	CR	8 m
Hwang et al. (40)	52/M	Pleural effusion	Multiple	NA	−	−	NA/56.3	+	47,XY, + add(5) (q11.2)x2,der(5;8) (q10;p10),del(7) (q32), t(15;17) (q22;q21)	*PML::RARA*	–	Favorable	ATRA + IDA	Dead	Died of shock and multi-organ failure
Oravcova et al. (41)	34/F	Left breast	multiple	MPO, CD34, Ki67 (60%∼70%)	+	−	3/NA	NA	Normal	*PML::RARA* (bcr3)	FLT3-ITD	Adverse	IDA + ATRA, intrathecal chemotherapy and CNS radiotherapy	CR	5 m and died of CNS failure
Collinge et al. (42)	49/F	Abdominal skin? purulent change	Multiple	MPO, CD68, CD163	+	−	NA/80	+	t(15;17)	*PML::RARA* (bcr1)	NA	Adverse	ATRA + ATO	CR	>6 m
Yamashita et al. (43)	50/M	(L2∼L4) extradural space? right rib? bones throughout body	Multiple	NA	NA	NA	−/NA	+	47,XY,+8, der(11;22) (q10;q10), add(14) (q32), der(15)t(15;17) (q22;q12), ider(17) (q10)t(15;17)*	*PML::RARA* *	NA		Before diagnosis of APL: radiotherapy, DA, HDAC, MA After diagnosis of APL: ATRA + DA (induction chemotherapy)? ATRA + ATO, GO + tamibarotene	CR	3 m and died of cerebral hemorrh-age
Ignacio-Cconchoy et al. (44)	35/M	Tongue	Single	MPO, CD68, CD15, Ki67 (88%)	+		NA/90	+	t(15;17) (q22;q21)	*PML::RARA*	NA	Adverse	ATRA + DNR	CR	NA
Wong et al. (45)	65/M	Heterogeneous allograft kidney	Single	MPO	NA	NA	0.02/NA	+	t(15;17) (q24;q21)	*PML::RARA*	NA	NA	–	–	Dead of cardiac arrest caused by coronary artery stenosis
Wang et al. (46)	77/F	Colon	Single	MPO, CD117, CD68, CK, CgA, Ki67 (65%)	−	−	68/NA	NA	t(15;17) (q22;q21)	*PML::RARA*	NA	Favorable	ATRA + ATO	CR	
Han et al. (47)	67/F	Right obturator internus, obturator externus and some lymph nodes	Multiple	NA	−	−	NA/72	NA	Normal	*CPSF6::* *RARG*	WT1, KRAS, EZH2	Favorable	ATRA + HA	NR	1 m and died of intracranial hemorrhage
Wang et al. (48)	2/F	Posterior fossa	Single	NA	−	−	NA/74.5	+	46,XX,t(4;17) (q12;q22)[ 9]/46, idem,del(16) (q22)[ 3]/45,idem,-x,-4,-9,-15,del(16) (q22), + marl, + mar2, + mar3[ 7]/46,xx[ 3]	*FIP1L1::* *RARA*	KRAS	Adverse	ATRA + DA	CR	5 m
Zhou and Li (49)	40/F	Right ovary (T9∼10) extradural space	Multiple	MPO, CD34, Lys	−	−	−/−	NA	46,XX[20]	*PML::RARA*	NA	Favorable	ATRA, right breast tumor excision, laminectomy	CR	288 m (but with relapse of MS at different sites)
Shu et al.(50)	50/F	(C6∼C7) extradural space	Multiple	MPO, TDT, CD56, CD43, Ki67 (60%)	−	−	−/50	−	t(15;17) (q24;q21)	*PML::RARA*	RUNX1, FLT3, KMT2C gene SNV and InDel	Favorable	Intraspinal tumor resection and spinal Galveston fixation, ATRA + ATO + DNR	CR	>10 m
Cho et al. (51)	56/M	extradural space	Multiple	MPO						*ZBTB16::* *RARA*					
Harrer et al. (52)	67/M	Right hemilarynx and skin	Multiple	CD45, MPO	−	−	50/NA	NA	Normal	*PML::RARA*	NA	Favorable	DA? ATRA, DA + ATRA, MA + ATRA	CR	>24 m
Loyaux et al. (53)	38/F	Pleural effusion	Multiple	CD45 dim, CD117, CD33, CD13	−	+	NA/NA	+	t(15;17) (q24;q21)	*PML::RARA* (bcr2) **	–	Favorable	ATRA + ATO	CR	NA (>2 m)
Chen et al. (54)	7/M	(L1) extradural space	Multiple	MPO, CD33	NA	NA	−/−	−	46,XY,dup(17) (q23q25)[15]/45,X,-Y,der(16)t(Y;16) (q12;q22), dup(17) (q23q25)[4]/45,X,-Y,del(4) (p14),der(16) t(Y;16) (q12; q22),dup(17) (q23q25)[1]	*TTMV::* *RARA*	WT1, SMAD9	NA	DAE (×6 cycles) + Ara-C (×4 cycles)	CR	6 m (and then MS relapse and BM infiltra-tion)

MS, myeloid sarcoma; WBC, white blood cell; DIC, disseminated intravascular coagulation; BM, bone marrow; PFS, progression-free survival; M, male; F, female; NA, not available; NR, non-remission; CR, complete remission; m, month(s); DA, daunorubicin and cytarabine; ATRA, all-trans-retinoic acid; HSCT, hematopoietic stem cell transplantation; IA or IDA, idarubicin and cytarabine; MA, melphalan and adriamycin; 6-MP, 6-mercaptopurine; MTX, methotrexate; DNR, daunorubicin; THP, pirarubicin; ATO, arsenic trioxide; HDAC, high-dose cytarabine; APL, acute promyelocytic leukemia; GO, gemtuzumab ozogamicin; HA, homoharringtonine and cytarabine; DAE, dexamethasone, cytarabine and etoposide. *Detected at second relapse of MS, not at initial diagnosis. **The fusion gene was detected only in pleural fluid and was negative in both blood and bone marrow.

A total of 40 patients received treatment, with 28 achieving remission, resulting in an overall response rate of 70%. Among low-risk patients, the remission rate was 70% (19 out of 27), while high-risk patients had a slightly higher remission rate of 83% (5 out of 6). Thirty patients were treated with ATRA combined with chemotherapy, and 83% (25 out of 30) achieved remission. One patient did not respond to treatment, two died from multiple organ failure, and two succumbed to intracranial hemorrhage. Additionally, three patients who received only chemotherapy also responded to treatment. Follow-up duration varied widely across studies, ranging from less than 1 week (20, 22) to as long as 288 months (49). This variation is attributed to several factors, including severe exhaustion and bleeding in some patients, either untreated or occurring during the myelosuppressive phase following surgery or chemotherapy. Notably, long-term survival was observed in patients who underwent surgery with careful monitoring and received ATRA combined with chemotherapy. Interestingly, the longest-followed patients experienced recurrent relapses of MS in various locations, despite no abnormalities being detected in the bone marrow or peripheral blood. These patients maintained long-term survival and good quality of life through surgery and chemotherapy. In addition to relapse at other extramedullary sites, MS/APL can progress to non-M3 AML. One patient, for instance, progressed to AML with the t(8;21)/RUNX1:RUNX1T1 translocation after more than 2 years of remission following ATRA and chemotherapy (25).

For patients with solitary MS and no bone marrow infiltration, the most common sites of infiltration were the spine (5 out of 17) and other bony sites (5 out of 17), followed by the pleura (2 out of 17). Singular cases were observed in the mediastinum, testis, ovary, and breast (1 each). Notably, no reports of involvement in digestive tract organs were found. Similar to other MS/APL cases, these patients were predominantly young, with a median age of 29 years, and there was no significant sex difference (male-to-female ratio of 10:7). The treatment response rate in this group was 70% (12 out of 17), with 91% (10 out of 11) of patients treated with ATRA achieving remission, while the remaining patients succumbed to severe bleeding. Five cases progressed to bone marrow infiltration. Three of these cases were thought to reflect disease development before treatment, while the remaining two cases developed bone marrow blasts or promyelocytes months after treatment initiation. This progression was considered to indicate a combination of disease relapse and progression.

## Discussion

Myeloid sarcoma typically manifests in patients with APL during relapse, with extramedullary involvement being relatively uncommon. It occurs in approximately 3%–5% of APL patients (12, 55). In the European multicenter PETHEMA trial, only 10 of 169 relapse cases showed extramedullary involvement, predominantly in the central nervous system and skin (14). Instances where MS presents as the initial symptom, without significant bone marrow or peripheral blood abnormalities, or where APL diagnostic criteria are not met, are exceedingly rare. Recent reports have identified new cases and additional sites of extramedullary infiltration. Among these, spinal extramedullary masses are the most frequently observed, followed by skin and pleura. Other tissues, including rarely transplanted tissue, have also been implicated. In MS/APL patients without evidence of disease in the bone marrow or peripheral blood, the extramedullary masses predominantly involve bony structures such as the spine, sternum, and humerus. However masses located in the digestive system are rare in these patients compared to other MS/APL cases. Therefore, isolated MS located in the skeleton is even more important to evaluate thoroughly and the possibility of promyelocytic sarcoma should be considered.

An important consideration for clinicians is the need for a comprehensive understanding and systematic evaluation of donor health in patients undergoing organ transplantation, to exclude blood-related diseases. In cases of MS/APL in transplanted organs, it is crucial to not only examine the patient’s bone marrow but also conduct *PML::RARA* testing on bone marrow and peripheral blood from both the donor and other recipients. This strategy ensures prompt detection and management of potential complications. The timing of detection post-transplant remains an unresolved issue that requires further investigation.

Clinicians often face significant challenges in diagnosing APL with MS, particularly when the presentation involves solitary promyelocytic sarcoma. When a mass is detected in any part of the body, fine needle aspiration often fails to provide sufficient diagnostic evidence of myeloid malignancy. In cases without coagulation abnormalities or other contraindications to surgery, a local pathological biopsy followed by immunohistochemical examination of the mass is essential to determine its origin. For suspected myeloid-origin tumors, it is critical to perform a bone marrow aspirate to rule out APL or other forms of non-M3 AML. Even when blood and bone marrow smears and flow cytometry do not show abnormalities, molecular testing is crucial. Both qPCR and FISH should be performed to detect *PML::RARA* fusion gene positivity. Although molecular analysis and FISH of MS biopsy tissue can be technically challenging, they are important for accurate diagnosis and should be performed whenever possible. For patients without atypical promyelocytes in the peripheral blood and bone marrow, and with no cytogenetic abnormalities, the detection of *PML::RARA* transcripts or *RARA* rearrangements in MS tissues via qPCR or FISH becomes the key diagnostic criterion. Additionally, karyotype analysis of the bone marrow, showing translocations involving chromosomes 15 and 17, can further strengthen diagnostic confidence in cases of solitary MS. Thus, the presence of *PML::RARA* is considered a critical marker for both the early diagnosis of solitary promyelocytic sarcoma and the monitoring of treatment efficacy and recurrence. An intriguing observation in some cases is the identification of rare fusion genes, although their association with MS/APL remains unclear. This highlights the need for further research to understand the significance of these rare fusions. Moreover, the absence of *PML::RARA* does not reliably exclude APL, emphasizing the importance of comprehensive testing. Next-generation sequencing and RT-PCR for other rare fusion transcripts could reveal unexpected findings, potentially offering new insights into MS/APL diagnostics.

This case is similar to previously reported MS/APL with a spinal intradural mass as the first manifestation, and the patient usually presents with low back pain and difficulty walking. These symptoms may occur with or without abnormal blood counts and coagulation. In this case, induction chemotherapy with ATRA combined with ATO was initiated after local lumpectomy. ATRA treatment continued to maintain *PML::RARA* negativity, followed by local radiotherapy. The patient achieved remission and maintained a good quality of life.

Patients with MS/APL, particularly those with spinal intraspinal masses at onset, often have a poor prognosis, highlighting the need for effective treatment strategies to improve outcomes. Treatment for these cases is similar to that for extramedullary relapses of APL, involving surgical decompression, local radiotherapy, and leukemia chemotherapy. Surgical resection is essential for reducing tumor volume, alleviating tissue compression, and preventing further spread. If coagulopathy is not significant, surgery should be performed promptly to relieve pain and improve mobility. Systemic therapy for the underlying leukemia is always necessary, regardless of bone marrow involvement or isolated MS/APL (56). ATRA, while effective, poorly penetrates the blood-brain barrier and is associated with relapses in the central nervous system (CNS) (57). Additionally, ATRA has been shown to increase tumor cell adhesion molecule expression (58–60), which could promote extramedullary metastasis and invasion. However, a higher incidence of extramedullary recurrence has not been observed in APL patients receiving ATRA compared to those treated with chemotherapy alone, though CNS recurrence is slightly more common, yet not statistically significant (55). Real-world data indicate that two-drug induction therapy combining ATRA and ATO offers longer disease-free survival compared to ATRA combined with chemotherapy (AIDA) (61–63). Thus, the combination of ATRA and ATO is recommended for treating *PML::RARA*-positive MS/APL. For rare *RARA* rearrangements, the specific fusion partners should be considered to determine whether ATRA is appropriate. The role of radiotherapy in treating APL-related extramedullary sarcoma remains debated. Some researchers view it as an effective strategy for eliminating residual tumor tissue and reducing recurrence risk after surgery (64, 65). However, others caution that local radiotherapy may increase the patient’s overall burden, leading to infections, treatment failure, or delays in chemotherapy (66). In some cases, patients intolerant to therapy have died from severe infections unrelated to chemotherapy. Furthermore, the potential for bone marrow infiltration by leukemic cells following radiotherapy, either from disease progression or radiotherapy-induced malignancy, remains a contentious issue. Given these considerations, we propose that a combination of ATRA and ATO be considered the optimal approach for treating *PML::RARA*-positive MS/APL. Local radiotherapy could be administered after consolidation therapy, weighing its potential benefits against its risks. New studies have explored the use of gilteritinib for extramedullary recurrence of APL with FLT3 mutations, showing rapid and sustained regression of the sarcoma (67). For patients with isolated MS/APL at initial diagnosis, whether targeted agents can improve remission and disease-free survival in the presence of specific gene mutations warrants further investigation. Additionally, hyperthermia, which shows synergistic effects with ATO in destabilizing *PML::RARA* fusion proteins both *in vivo* and *in vitro*, may offer a promising new therapeutic strategy (68).

## Conclusion

In conclusion, we describe the rare presentation of APL solely as MS in a patient, which ultimately led to the diagnosis of MS/APL. Additionally, we provide a comprehensive review of similar cases to further elucidate this uncommon clinical manifestation of APL. The case and literature review contribute to the growing body of knowledge regarding the presentation, diagnosis, and treatment of MS/APL, potentially guiding future clinical practice in similar cases.

## References

[1] Jimenez JJ Chale RS Abad AC Schally AV. Acute promyelocytic leukemia (APL): A review of the literature. *Oncotarget.* (2020) 11:992–1003. 32215187 10.18632/oncotarget.27513 PMC7082115

[2] Mannan A Muhsen IN Barragán E Sanz MA Mohty M Hashmi SK Genotypic and phenotypic characteristics of acute promyelocytic leukemia translocation variants. *Hematol Oncol Stem Cell Ther.* (2020) 13:189–201. 10.1016/j.hemonc.2020.05.007 32473106

[3] Hillestad LK. Acute promyelocytic leukemia. *Acta Med Scand.* (1957) 159:189–94. 13508085

[4] Pui MH Fletcher BD Langston JW. Granulocytic sarcoma in childhood leukemia: Imaging features. *Radiology.* (1994) 190:698–702. 8115614 10.1148/radiology.190.3.8115614

[5] Tomita A Kiyoi H Naoe T. Mechanisms of action and resistance to all-trans retinoic acid (ATRA) and arsenic trioxide (As2O 3) in acute promyelocytic leukemia. *Int J Hematol.* (2013) 97:717–25. 10.1007/s12185-013-1354-4 23670176

[6] Huang ME Ye YC Chen SR Chai JR Lu JX Zhoa L Use of all-trans retinoic acid in the treatment of acute promyelocytic leukemia. *Blood.* (1988) 72:567–72. 3165295

[7] Magdy M Abdel Karim N Eldessouki I Gaber O Rahouma M Ghareeb M Myeloid sarcoma. *Oncol Res Treat.* (2019) 42:224–9. 30840960 10.1159/000497210

[8] Almond LM Charalampakis M Ford SJ Gourevitch D Desai A. Myeloid sarcoma: Presentation, diagnosis, and treatment. *Clin Lymphoma Myeloma Leuk.* (2017) 17:263–7. 28342811 10.1016/j.clml.2017.02.027

[9] Ooi GC Chim CS Khong PL Au WY Lie AK Tsang KW Radiologic manifestations of granulocytic sarcoma in adult leukemia. *Am J Roentgenol.* (2001) 176:1427–31. 11373207 10.2214/ajr.176.6.1761427

[10] Guermazi A Feger C Rousselot P Merad M Benchaib N Bourrier P Granulocytic sarcoma (chloroma): Imaging findings in adults and children. *Am J Roentgenol.* (2002) 178:319–25. 11804886 10.2214/ajr.178.2.1780319

[11] Benekli M Savaş MC Haznedaroğlu IC Dündar SV. Granulocytic sarcoma in acute promyelocytic leukemia. *Leuk Lymphoma.* (1996) 22:183–6. 8724548 10.3109/10428199609051748

[12] Albano F Specchia G. Extramedullary disease in acute promyelocytic leukemia: Two-in-one disease. *Mediterr J Hematol Infect Dis.* (2011) 3:e2011066. 10.4084/MJHID.2011.066 22220263 PMC3248343

[13] Pileri SA Ascani S Cox MC Campidelli C Bacci F Piccioli M Myeloid sarcoma: Clinico-pathologic, phenotypic and cytogenetic analysis of 92 adult patients. *Leukemia.* (2007) 21:340–50. 10.1038/sj.leu.2404491 17170724

[14] de Botton S Sanz MA Chevret S Dombret H Martin G Thomas X Extramedullary relapse in acute promyelocytic leukemia treated with all-trans retinoic acid and chemotherapy. *Leukemia.* (2006) 20:35–41. 16307026 10.1038/sj.leu.2404006

[15] Tosi A De Paoli A Fava S Luoni M Sironi M Tocci A Undifferentiated granulocytic sarcoma: A case with epidural onset preceding acute promyelocytic leukemia. *Haematologica.* (1995) 80:44–6. 7758990

[16] Belasco JB Bryan JH McMillan CW. Acute promyelocytic leukemia presenting as a pelvic mass. *Med Pediatr Oncol.* (1978) 4:289–95. 10.1002/mpo.2950040403 281594

[17] Kubonishi I Ohtsuki Y Machida K Agatsuma Y Tokuoka H Iwata K Granulocytic sarcoma presenting as a mediastinal tumor. Report of a case and cytological and cytochemical studies of tumor cells in vivo and in vitro. *Am J Clin Pathol.* (1984) 82:730–4. 10.1093/ajcp/82.6.730 6594928

[18] Zuiable A Aboud H Nandi A Powles R Treleaven J. Extramedullary disease initially without bone marrow involvement in acute promyelocytic leukaemia. *Clin Lab Haematol.* (1989) 11:288–9. 2591162 10.1111/j.1365-2257.1989.tb00223.x

[19] Bobbio-Pallavicini E Cannatelli G Motta E Grassi M Bergamaschi G Rosso R Histologic diagnosis and precocious treatment in a case of isolated promyelocytic sarcoma. *Leukemia.* (1998) 12:2035–6. 10.1038/sj.leu.2401227 9844936

[20] Takeh H Farran M Debaize JP. Granulocytic sarcoma (chloroma) of the small intestine. *Acta Chir Belg.* (1999) 99:78–81. 10352737

[21] Gopal S Marcussen S Dobin SM Koss W Donner LR. Primary myeloid sarcoma of the testicle with t(15;17). *Cancer Genet Cytogenet.* (2005) 157:148–50. 10.1016/j.cancergencyto.2004.06.010 15721636

[22] Fukushima S Terasaki M Tajima Y Shigemori M. Granulocytic sarcoma: An unusual complication of acute promyelocytic leukemia causing cerebellar hemorrhage. Case report. *J Neurosurg.* (2006) 105:912–5. 10.3171/jns.2006.105.6.912 17405265

[23] Worch J Ritter J Frühwald MC. Presentation of acute promyelocytic leukemia as granulocytic sarcoma. *Pediatr Blood Cancer.* (2008) 50:657–60. 17437290 10.1002/pbc.21190

[24] Mohamedbhai S Pule M Conn B Hopper C Ramsay A Khwaja A Acute promyelocytic leukaemia presenting with a myeloid sarcoma of the tongue. *Br J Haematol.* (2008) 141:565. 10.1111/j.1365-2141.2008.07080.x 18373707

[25] Wang X Liu H Wu Z Xu X Chen X Zhai Z A case of acute promyelocytic leukemia presenting with a nonleukemic granulocytic sarcoma of the ovary, with subsequent development of acute myeloid leukemia associated with t(8;21). *Leuk Res.* (2009) 33:580–2. 10.1016/j.leukres.2008.08.008 18804280

[26] Thomas X Chelghoum Y. Promyelocytic sarcoma of the sternum: A case report and review of the literature. *Korean J Hematol.* (2011) 46:52–6. 10.5045/kjh.2011.46.1.52 21461306 PMC3065629

[27] Kyaw TZ Maniam JA Bee PC Chin EF Nadarajan VS Shanmugam H Myeloid sarcoma: An unusual presentation of acute promyelocytic leukemia causing spinal cord compression. *Turk J Haematol.* (2012) 29:278–82. 10.5505/tjh.2012.94809 24744674 PMC3986755

[28] Bittencourt H Teixeira Junior AL Glória AB Ribeiro AF Fagundes EM. Acute promyelocytic leukemia presenting as an extradural mass. *Rev Bras Hematol Hemoter.* (2011) 33:478–80. 10.5581/1516-8484.20110126 23049367 PMC3459371

[29] Shvartsbeyn M Pandey S Mercer SE Goldenberg G. Leukemia cutis presenting clinically as disseminated herpes zoster in a patient with unrecognized acute promyelocytic leukemia. *J Clin Aesthet Dermatol.* (2012) 5:40–3 22708008 PMC3366444

[30] Benjazia E Khalifa M Benabdelkader A Laatiri A Braham A Letaief A Granulocytic sarcoma of the rectum: Report of one case that presented with rectal bleeding. *World J Gastrointest Pathophysiol.* (2010) 1:144–6. 10.4291/wjgp.v1.i4.144 21607155 PMC3097956

[31] Damodar S Prashantha B Gangoli A Gopalakrishnan G Jayanthi KJ. Granulocytic sarcoma of colon in a patient with acute promyelocytic leukemia. *Indian J Hematol Blood Transfus.* (2013) 29:152–4. 10.1007/s12288-012-0152-0 24426361 PMC3710555

[32] Yamashita Y Isomura N Hamasaki Y Goto M. Case of pediatric acute promyelocytic leukemia presenting as extramedullary tumor of the mandible. *Head Neck.* (2013) 35:E310–3. 10.1002/hed.23163 22972688

[33] Piñán MA Ardanaz MT Guinea JM García-Ruiz JC. Myeloid sarcoma preceding an acute promyelocytic leukaemia with neuromeningeal infiltration. *Ann Hematol.* (2014) 93:339–40. 10.1007/s00277-013-1795-0 23716188

[34] Li J Tu C Wang D Huang C Zhang X. [Myeloid sarcoma with acute promyelocytic leukemia: Two cases report]. *Zhonghua Xue Ye Xue Za Zhi.* (2015) 36:438–40. 26031537 10.3760/cma.j.issn.0253-2727.2015.05.020 PMC7342602

[35] Kikuma T Nakamachi Y Noguchi Y Okazaki Y Shimomura D Yakushijin K A new transcriptional variant and small azurophilic granules in an acute promyelocytic leukemia case with NPM1/RARA fusion gene. *Int J Hematol.* (2015) 102:713–8. 26342691 10.1007/s12185-015-1857-2

[36] Rodriguez EA Lopez MA Valluri K Wang D Fischer A Perdomo T Acute appendicitis secondary to acute promyelocytic leukemia. *Am J Case Rep.* (2015) 16:73–6. 25666852 10.12659/AJCR.892760 PMC4327184

[37] Shah NN Stonecypher M Gopal P Luger S Bagg A Perl A Acute promyelocytic leukemia presenting as a paraspinal mass. *J Commun Support Oncol.* (2016) 14:126–9. 10.12788/jcso.0220 27058871

[38] de Andrade BA Farneze RB Agostini M Cortezzi EB Abrahão AC Cabral MG Myeloid sarcoma of the oral cavity: A case report and review of 89 cases from the literature. *J Clin Exp Dent.* (2017) 9:e1167–71. 10.4317/jced.53935 29075423 PMC5650223

[39] Sawhney S Holtzman NG Davis DL Kaizer H Giffi V Emadi A Promyelocytic sarcoma of the right humerus: An unusual clinical presentation with unique diagnostic and treatment considerations. *Clin Case Rep.* (2017) 5:1874–7. 10.1002/ccr3.1212 29152290 PMC5676285

[40] Hwang N Roh S Ham JY Suh JS. Leukemic pleural effusion in acute promyelocytic leukemia: A case report. *Lab Med Online.* (2018) 8:24–8.

[41] Oravcova I Mikuskova E Leitnerova M Gyarfas J Mlcakova A Szepe P A unique clinical presentation of de novo acute promyelocytic leukemia as a myeloid sarcoma of the breast. *Int J Hematol.* (2018) 108:550–3. 10.1007/s12185-018-2479-2 29931624

[42] Collinge E Tigaud I Balme B Gerland LM Sujobert P Carlioz V Case report: Purulent transformation of granulocytic sarcoma: An unusual pattern of differentiation in acute promyelocytic leukemia. *Medicine (Baltimore).* (2018) 97:e9657. 10.1097/MD.0000000000009657 29465554 PMC5841987

[43] Yamashita T Nishijima A Noguchi Y Narukawa K Oshikawa G Takano H Acute promyelocytic leukemia presenting as recurrent spinal myeloid sarcomas 3 years before developing leukemia: A case report with review of literature. *Clin Case Rep.* (2019) 7:316–21. 10.1002/ccr3.1991 30847197 PMC6389481

[44] Ignacio-Cconchoy FL Benites-Zapata VA Yanac-Avila RL Vela-Velàsquez CT. Myeloid sarcoma of the tongue as a first manifestation of acute promyelocytic leukemia: A case report. *Rep Pract Oncol Radiother.* (2020) 25:174–7. 10.1016/j.rpor.2019.12.026 32021572 PMC6994280

[45] Wong RL Ketcham M Irwin T Akilesh S Zhang TY Reyes JD Donor-derived acute promyelocytic leukemia presenting as myeloid sarcoma in a transplanted kidney. *Leukemia.* (2020) 34:2776–9. 10.1038/s41375-020-0903-0 32523036 PMC7515823

[46] Wang L Cai DL Lin N. Myeloid sarcoma of the colon as initial presentation in acute promyelocytic leukemia: A case report and review of the literature. *World J Clin Cases.* (2021) 9:6017–25. 10.12998/wjcc.v9.i21.6017 34368322 PMC8316963

[47] Han X Jin C Zheng G Li Y Wang Y Zhang E Acute myeloid leukemia with CPSF6-RARG fusion resembling acute promyelocytic leukemia with extramedullary infiltration. *Ther Adv Hematol.* (2021) 12:2040620720976984. 10.1177/2040620720976984 33473264 PMC7797573

[48] Wang Y Rui Y Shen Y Li J Liu P Lu Q Myeloid sarcoma type of acute promyelocytic leukemia with a cryptic insertion of RARA into FIP1L1: The clinical utility of NGS and bioinformatic analyses. *Front Oncol.* (2021) 11:688203. 10.3389/fonc.2021.688203 34249738 PMC8264125

[49] Zhou X Li C. Long-term survival in an acute promyelocytic leukemia patient with recurrent granulocytic sarcomas: A case report. *Medicine (Baltimore).* (2021) 100:e25257. 10.1097/MD.0000000000025257 33832087 PMC8036079

[50] Shu X Wu Q Guo T Yin H Liu J. Acute promyelocytic leukemia presenting with a myeloid sarcoma of the spine: A case report and literature review. *Front Oncol.* (2022) 12:851406. 10.3389/fonc.2022.851406 35311073 PMC8931201

[51] Cho EJ Byeon SJ Hyun J Kim HS Jung JY. A ZBTB16-RARα variant of acute promyelocytic leukemia with concurrent myeloid sarcoma presenting as sudden onset paraplegia. *Clin Lab.* (2022) 68:1963–6. 10.7754/Clin.Lab.2021.211227 36125151

[52] Harrer DC Lüke F Einspieler I Menhart K Hellwig D Utpatel K Case report: Extramedullary acute promyelocytic leukemia: An unusual case and mini-review of the literature. *Front Oncol.* (2022) 12:886436. 10.3389/fonc.2022.886436 35692786 PMC9174987

[53] Loyaux R Lecolant S Cysique Foilan L Pradon C Cotteret S Micol JB An atypical promyelocytic sarcoma and pleural effusion in a patient with Gorham’s disease: Efficiency of ATRA/ATO-based treatment. *Clin Case Rep.* (2023) 11:e7785. 10.1002/ccr3.7785 37601428 PMC10432579

[54] Chen J Zhou X Wang Y Zhang Y Chen X Wang T TTMV::RARA-driven myeloid sarcoma in pediatrics with germline SAMD9 mutation and relapsed with refractory acute promyelocytic leukemia. *Int J Lab Hematol.* (2024) 46:190–4. 10.1111/ijlh.14189 37855178

[55] Specchia G Lo Coco F Vignetti M Avvisati G Fazi P Albano F Extramedullary involvement at relapse in acute promyelocytic leukemia patients treated or not with all-trans retinoic acid: A report by the Gruppo Italiano Malattie Ematologiche dell’Adulto. *J Clin Oncol.* (2001) 19:4023–8. 11600603 10.1200/JCO.2001.19.20.4023

[56] Bakst R Powers A Yahalom J. Diagnostic and therapeutic considerations for extramedullary leukemia. *Curr Oncol Rep.* (2020) 22:75. 10.1007/s11912-020-00919-6 32577912

[57] Ferreira R Napoli J Enver T Bernardino L Ferreira L. Advances and challenges in retinoid delivery systems in regenerative and therapeutic medicine. *Nat Commun.* (2020) 11:4265. 10.1038/s41467-020-18042-2 32848154 PMC7450074

[58] Marchetti M Falanga A Giovanelli S Oldani E Barbui T. All-trans-retinoic acid increases adhesion to endothelium of the human promyelocytic leukaemia cell line NB4. *Br J Haematol.* (1996) 93:360–6. 8639429 10.1046/j.1365-2141.1996.4911029.x

[59] Saiki I Fujii H Yoneda J Abe F Nakajima M Tsuruo T Role of aminopeptidase N (CD13) in tumor-cell invasion and extracellular matrix degradation. *Int J Cancer.* (1993) 54:137–43. 8097496 10.1002/ijc.2910540122 PMC7165932

[60] Brown DC Tsuji H Larson RS. All-trans retinoic acid regulates adhesion mechanism and transmigration of the acute promyelocytic leukaemia cell line NB-4 under physiologic flow. *Br J Haematol.* (1999) 107:86–98. 10.1046/j.1365-2141.1999.01671.x 10520028

[61] Lo-Coco F Avvisati G Vignetti M Thiede C Orlando SM Iacobelli S Retinoic acid and arsenic trioxide for acute promyelocytic leukemia. *N Engl J Med.* (2013) 369:111–21. 23841729 10.1056/NEJMoa1300874

[62] Kulkarni UP Selvarajan S Lionel S Prakash MA Palani HK Balasundaram N Real world data with concurrent retinoic acid and arsenic trioxide for the treatment of acute promyelocytic leukemia. *Blood Cancer J.* (2022) 12:22. 10.1038/s41408-022-00619-3 PMC8803919 35102152

[63] Voso MT Guarnera L Lehmann S Döhner K Döhner H Platzbecker U Acute promyelocytic leukemia: Long-term outcomes from the HARMONY project. *Blood.* (2024). [Epub ahead of print]. 10.1182/blood.2024026186 39504485

[64] Song JH Son SH Lee JH Chung SM Jang HS Choi BO Defining the optimal dose of radiation in leukemic patients with extramedullary lesions. *BMC Cancer.* (2011) 11:428. 10.1186/1471-2407-11-428 21975070 PMC3196724

[65] Graham SR. Treatment of extramedullary myeloid sarcoma with radiotherapy. *Cureus.* (2021) 13:e15676. 10.7759/cureus.15676 PMC8281801 34277267

[66] Salerno KE. Radiation therapy for soft tissue sarcoma: Indications, timing, benefits, and consequences. *Surg Clin North Am.* (2022) 102:567–82. 35952688 10.1016/j.suc.2022.04.001 PMC9372474

[67] Hou CX Chen Y Liu SH Jiang YZ Huang DP Chen SN Effective treatment with Gilteritinib-based regimens for FLT3-mutant extramedullary relapse in acute promyelocytic leukemia. *Hematology.* (2024) 29:2293496. 10.1080/16078454.2023.2293496 38095349

[68] Maimaitiyiming Y Wang QQ Yang C Ogra Y Lou Y Smith CA Hyperthermia selectively destabilizes oncogenic fusion proteins. *Blood Cancer Discov.* (2021) 2:388–401. 10.1158/2643-3230.BCD-20-0188 34661159 PMC8513904

